# Draft Genome Sequence of *Terasakiispira papahanaumokuakeensis* PH27A^T^, a Spiral Bacterium from the Northwestern Hawaiian Islands

**DOI:** 10.1128/genomeA.01166-16

**Published:** 2016-10-20

**Authors:** Xuehua Wan, Luis Dasilveira, Shaobin Hou, Jennifer A. Saito, Stuart P. Donachie

**Affiliations:** a Advanced Studies in Genomics, Proteomics and Bioinformatics, University of Hawai‘i at Mānoa, Honolulu, Hawai‘i, USA; b Department of Microbiology, University of Hawai‘i at Mānoa, Honolulu, Hawai‘i, USA

## Abstract

The genus *Terasakiispira* hosts only *Terasakiispira papahanaumokuakeensis* PH27A^T^, cultivated from an anchialine pond on Pearl and Hermes Atoll, Northwestern Hawaiian Islands. The strain’s genome sequence may provide insights into the evolution of free-living *Oceanospirillaceae*.

## GENOME ANNOUNCEMENT

The genus *Terasakiispira* was established to accommodate strain PH27A^T^, the sole strain of *Terasakiispira papahanaumokuakeensis* (1, 2). PH27A^T^ cells are spiral and motile by bipolar tufts of flagella. The strain shares 93.3 and 92.1% nucleotide identity in the 16S rRNA gene with its nearest-described neighbors, *Marinospirillum celere* v1c_Sn-red^T^ and *M. alkaliphilum* Z4^T^, respectively. PH27A^T^ was distinguished from *Marinospirillum* species by its hydrolysis of gelatin and the abundances of ubiquinone Q-9 and cadaverine (1, 2). Cells of PH27A^T^ accumulate poly-β-hydroxybutyrate (PHB), a biodegradable polymer that has potential applications in medicine and the food industry. We present here the draft genome sequence of *T. papahanaumokuakeensis* PH27A^T^.

Genomic DNA was isolated from a 48-h culture of PH27A^T^ in marine broth 2216E (Difco) using the Wizard genomic DNA purification kit (Promega, USA), with cetyltrimethylammonium bromide (CTAB) and phenol extraction steps. We generated 123.6 Mb of shotgun sequences and 84.3 Mb of 8-kb-span paired-end sequences using the Roche 454 GS FLX+ Titanium pyrosequencing platform, providing ~50× genome coverage. Newbler 2.8 built two scaffolds totaling 4,048,029 bp (scaffold *N*_50_, 4,042,489 bp), comprising 22 contigs (contig *N*_50_, 404.2 kb); 99.99% of the assembled bases have a base accuracy quality value above the Phred quality score of Q40. The genome’s G+C content is 51.3%.

The genome was annotated in the NCBI Prokaryotic Genome Annotation Pipeline (PGAP) and Rapid Annotation using Subsystem Technology (RAST) (3–5). PGAP identified 3,404 protein-coding genes, 62 tRNA coding regions, and 5 rRNA coding regions. RAST identified 3,647 protein-coding open reading frames, 62 tRNA coding regions, and 480 subsystems. RAST identified the flagellar assembly system and chemotaxis system in PH27A^T^, consistent with its formal description (1). Thirty-seven metallopeptidases were predicted by BLASTP against the MEROPS database 10.0 (6). Homologs of three enzymes (acetyl-coenzyme A [acetyl-CoA] acetyltransferase, acetoacetyl-CoA reductase, and poly-β-hydroxybutyrate polymerase) in the PHB biosynthetic pathway were predicted in the PH27A^T^ proteome with BLASTP, as were three enzymes listed in the MetaCyc database (*E* < 1e^−10^) (7). Further data mining of the PH27A^T^ genome will shed light on the physiological characteristics and evolutionary features that distinguish PH27A^T^ and this genus from neighboring *Oceanospirillaceae* and other *Gammaproteobacteria*.

### Accession number(s).

This whole-genome shotgun project has been deposited at DDBJ/EMBL/GenBank under accession number MDTQ00000000. The version described in this paper is the first version, MDTQ01000000.
